# Current Evidence-Based Treatment of Angina With Nonobstructive Coronary Arteries (ANOCA)

**DOI:** 10.1016/j.jscai.2025.102633

**Published:** 2025-04-15

**Authors:** Olga Toleva, Nathaniel R. Smilowitz, Odayme Quesada, Manoj Kesarwani, Michael P. Savage, Megha Prasad, Ailin Barseghian El-Farra, Noa Holoshitz, Jeffrey W. Moses, William F. Fearon, Jennifer A. Tremmel, Bruce Samuels, Timothy D. Henry, Microvascular Network

**Affiliations:** a Georgia Heart Institute, Northeast Georgia Medical Centre, Gainesville, Georgia; b Division of Cardiology, Department of Medicine, New York University Grossman School of Medicine, NYU Langone Health, New York, New York; c Cardiology Section, Department of Medicine, VA New York Harbor Healthcare System, New York, New York; d Women’s Heart Center, Heart & Vascular Institute, The Christ Hospital Health Network, Cincinnati, Ohio; e Department of Internal Medicine, University of Cincinnati, Cincinnati, Ohio; f Department of Internal Medicine, Division of Cardiovascular Medicine, University of California, Davis School of Medicine, Sacramento, California; g Department of Medicine, Sidney Kimmel Medical College, Thomas Jefferson University Hospital, Philadelphia, Pennsylvania; h Division of Cardiology, NewYork Presbyterian Hospital/Columbia University Irving Medical Center, New York, New York; i Department of Medicine, Division of Cardiology, University of California, Irvine, California; j Ascension Wisconsin, Columbia St Mary's Hospital, Milwaukee, Wisconsin; k St. Francis Hospital & Heart Center, Roslyn, New York; l Division of Cardiovascular Medicine and the Stanford Cardiovascular Institute, Stanford University School of Medicine, Stanford, California; m VA Palo Alto Health Care System, Palo Alto, California; n Smidt Heart Institute, Cedars-Sinai Medical Center, Los Angeles, California; o The Lindner Research Center at The Christ Hospital, Cincinnati, Ohio

**Keywords:** angina with nonobstructive coronary arteries, coronary microvascular dysfunction, coronary spasm, microvascular angina, spasm, treatment, myocardial bridge

## Abstract

Although the techniques for coronary function testing (CFT) were largely developed more than 30 years ago, consensus on the specific diagnostic criteria and endotypes is still lacking. Furthermore, the management of patients with angina with nonobstructive coronary arteries (ANOCA) is challenging for most cardiologists. These patients are often a burden to the health care system due to recurrent presentations to the emergency room, repeated coronary angiography, and lack of improvement in their anginal symptoms even after being properly diagnosed with CFT. Therapies for coronary microvascular dysfunction, coronary vasospasm, and myocardial bridging have been tested in small trials and only a few randomized controlled trials. However, we are still limited in the options of therapies that we can offer to our patients. Although techniques and principles of CFT have advanced significantly, the art of managing patients with ANOCA is very challenging. A dedicated comprehensive summary of all contemporary treatment options for ANOCA is needed to guide clinicians in managing this challenging population of patients. This article subdivides ANOCA into 3 major categories: coronary microvascular dysfunction, coronary vasospasm, and myocardial bridging. Patients with ANOCA may present with 1 or a combination of the described categories. We describe major pillars in managing ANOCA patients: risk factors and lifestyle changes, traditional pharmacotherapy, device therapies, and novel approaches. We offer a summary of most evidence-based trials and scientific data on therapies for patients with ANOCA. This summary of available data about therapies aims to improve practitioners' knowledge and give more scientific merit to the care of patients with ANOCA.

## Introduction

Nearly half of patients with stable angina and/or ischemia do not have obstructive epicardial coronary disease.^1^ Angina with nonobstructive coronary arteries (ANOCA) has several underlying mechanisms that can be determined by invasive coronary function testing (CFT), including coronary microvascular dysfunction (CMD), microvascular spasm, endothelial dysfunction, epicardial coronary vasospasm, or myocardial bridging.^2^, ^3^, ^4^, ^5^ Patients with ANOCA are best managed with therapies specific to the underlying disorder.^2^^,^^4^, ^5^, ^6^

Clinical practice guidelines recognize the clinical impact of ANOCA and recommend CFT to define ANOCA endotypes and appropriate treatment.^7^^,^^8^ In some instances, patients previously treated for obstructive coronary artery disease (CAD) may have persistent angina related to unrecognized CMD, vasospasm, or myocardial bridging, despite successful epicardial coronary revascularization. Optimal medical treatment for these conditions remains challenging due to the potential for multiple superimposed mechanisms and the absence of large, randomized controlled trials (RCT) assessing treatments. We review the existing evidence supporting therapies for patients with ANOCA, with a focus on the treatment of CMD, coronary vasospasm, and myocardial bridging.

## Risk factor and lifestyle modification strategies in ANOCA

Lifestyle interventions should be instituted in all patients with ANOCA. However, data are limited. An RCT of 62 women testing multiple lifestyle interventions including intensified risk factor control, a low-calorie diet, and an exercise training program, led to reductions in angina frequency and weight loss but did not increase coronary flow reserve (CFR).^9^ Cognitive behavioral therapy can improve angina symptoms and reduce recurrent episodes within the first 3 months of treatment.^10^ Meditation can also improve quality of life (QoL) and reduce angina in ANOCA.^11^^,^^12^

## Pharmacotherapy

### Strategy trials

Few contemporary randomized trials provide evidence-based management strategies specific to the ANOCA endotype. The CorMicA trial enrolled 151 individuals with ANOCA who underwent blinded CFT and were randomly assigned to receive therapy based on results of testing versus usual care with empiric treatment (without knowledge of the CFT results).^6^ Patients with cardiovascular risk factors, including hyperlipidemia and hypertension were considered for baseline therapy with aspirin, statin, and angiotensin-converting enzyme inhibitors (ACEi). In the intervention arm, participants with CMD or microvascular spasm (defined as microvascular angina in the trial) were advised to receive a nonselective beta blocker, such as carvedilol 6.25 mg twice a day or nebivolol 2.5 mg, as the first-line antianginal agent. Calcium channel blockers (CCB), such as verapamil 40 mg twice a day, were suggested as second-line therapy, to be added or substituted when beta-blockers were ineffective or not tolerated. Dihydropyridine (DHP) CCB, nicorandil, or ranolazine were recommended as third-line antianginal therapies. Patients with epicardial vasospasm were advised to receive CCB and long-acting nitrates (LAN) as first-line therapy, in addition to aspirin and statin if atherosclerosis or endothelial dysfunction were present. At baseline, QoL and angina summary scores were similar between the 2 groups, but at 1 year, patients assigned to targeted therapy had better scores than participants assigned to usual care with empiric therapy.^6^ CorMicA was the first RCT to provide evidence that stratified medical therapy based on CFT improves QoL and reduces angina burden in patients with ANOCA. A larger multicenter study assessing the impact of CFT on QoL is currently ongoing.

The ongoing strategy-based WARRIOR trial is a large, prospective, multicenter, RCT of women with ischemia and nonobstructive CAD who were randomly assigned to either an intensive medical strategy with a high-intensity statin, ACEi or angiotensin receptor blockers (ARB), and low-dose aspirin versus usual care. The primary end point is all-cause mortality and nonfatal cardiovascular events.

Until rigorous trials are conducted to define optimal treatments for specific underlying mechanisms of ANOCA, treatment strategies are largely informed by studies enrolling participants with heterogeneous diseases.

### Traditional antianginal pharmacotherapy for ANOCA

#### *Beta-*blockers

##### CMD

Beta-adrenergic receptor blockers remain a cornerstone of modern CMD treatment despite relatively limited evidence of efficacy. Most trials have been small and include heterogeneous cohorts of patients with ANOCA. Beta-blockers reduced angina, improved QoL, and extended the time to ST-segment depression during stress testing more than placebo, LAN, and CCB in a randomized crossover trial of 10 patients with undifferentiated ANOCA.^13^ In an observational study of 51 patients with presumed microvascular angina who were prescribed antianginal therapy, a greater proportion of those with improved QoL were prescribed beta blocker therapy (88% vs 27%, *P* < .001) versus individuals without improvements in QoL.^14^ Nonselective beta-blockers (eg, carvedilol) or beta-blockers with nitric oxide (NO)-mediated vasodilatory effects (eg, nebivolol) may be preferred to improve coronary blood flow (CBF) in patients with CMD.^15^, ^16^, ^17^, ^18^ However, in a double-blind RCT of nebivolol 10 mg versus atenolol 100 mg in 24 patients with ANOCA, no differences in Doppler-derived CFR, hyperemic microvascular resistance, or response to acetylcholine (Ach) were observed at 1 year of treatment.^17^ In contrast, in a trial of 30 patients with ANOCA randomly assigned to nebivolol 5 mg daily or metoprolol 50 mg daily for 12 weeks, nebivolol prolonged exercise duration and the time to ST-segment depression, and increased the proportion of patients with improvements in Canadian Cardiovascular Society (CCS) angina classification compared with metoprolol.^19^

##### Vasospasm

Although beta-blockers are commonly prescribed for CMD, they should be used with caution or avoided in individuals with concomitant vasospasm due to the potential for unopposed alpha-adrenergic activity that can enhance coronary vasomotor tone. In a study of propranolol monotherapy in patients with well-characterized vasospasm, beta-blockers prolonged the duration of anginal episodes but did not increase their frequency.^20^

##### Myocardial bridging

Beta-blockers are first-line therapy for myocardial bridging due to their negative chronotropic and inotropic effects to enhance diastolic coronary perfusion and reduce systolic coronary compression and myocardial oxygen demand. Among individuals with myocardial bridges and concomitant endothelial dysfunction or vasospasm, nebivolol can be considered for its additional endothelium-dependent vasodilatory properties. However, evidence for beta blocker use in individuals with myocardial bridges is limited to observational studies.^21^

#### Calcium Channel Blockers

##### CMD

The role of CCB as therapy for CMD is uncertain. In a randomized crossover trial of 26 individuals with ANOCA and CMD, CCB was associated with improved exercise duration, fewer anginal episodes, and less nitroglycerin use compared with placebo.^22^ In a study of 68 patients with ANOCA, a combination of daily CCB (diltiazem 90 mg) and statin (fluvastatin 40 mg) maximally improved CFR by echocardiography, increased exercise time to ST-segment depression, increased NO, and reduced endothelin-1.^23^ However, when CCB were compared with beta-blockers and LAN in a randomized crossover trial enrolling 10 patients with ANOCA, only beta-blockers improved anginal symptoms.^13^ Multiple studies suggest that CCB do not improve invasive measurements of CFR in patients with CMD. The EDIT-CMD trial tested the effect of diltiazem in 126 patients with ANOCA.^24^ All patients underwent CFT to diagnose vasospasm by Ach provocation and/or CMD by adenosine CFR <2.0 and/or index of microcirculatory resistance (IMR) ≥25. Patients with vasospasm or abnormal CFR/IMR were then randomly assigned to receive diltiazem up to 360 mg daily versus placebo, and 73 patients underwent repeat invasive CFT after 6 weeks of treatment.^24^ Diltiazem did not result in any improvement in symptoms, QoL, or microvascular parameters, but did reduce the prevalence of epicardial spasm. In the randomized crossover ChaMP-CMD trial, patients with CMD, defined as those with a CFR<2.5, had improvements in exercise time and modest improvements in angina scores after 4 weeks of daily amlodipine.^25^

##### Vasospasm

Dihydropyridine and non-DHP CCB are recommended as first-line therapies for managing angina due to vasospasm, based on the results of multiple placebo-controlled RCT.^26^, ^27^, ^28^, ^29^ In a trial of 51 hypertensive patients with vasospasm randomly assigned to diltiazem, nebivolol, or a combination of the 2, all therapies reduced diameter narrowing with invasive Ach provocation testing, but the greatest improvements were observed in the diltiazem-only group.^30^ The optimal CCB to treat vasospasm is uncertain. Sustained release (diltiazem R 100 mg twice daily) and long-acting (nifedipine CR 40 mg once a day) CCB were equally effective in alleviating angina in a small RCT of patients with vasospasm.^31^ In refractory cases of coronary vasospasm, a combination of non-DHP and DHP CCB may provide synergistic effects.^32^ In observational studies of patients with vasospasm, benidipine was associated with reduced anginal symptoms compared with diltiazem, and a more favorable prognosis versus other CCB.^33^, ^34^, ^35^ However, in a small RCT of 30 patients with vasospasm who were randomly assigned to nifedipine CR 40 mg daily versus benidipine 4 mg twice daily, the nifedipine group had fewer anginal episodes and less nitroglycerin after 8 weeks of follow-up.^36^ Currently, benidipine is only available in Asia. In the EDIT-CMD trial of diltiazem versus placebo, diltiazem resolved epicardial spasms in 47% of affected patients during repeat Ach provocation testing. However, in 31%, the epicardial spasm was replaced by microvascular spasm, and only 16% had no spasm at follow-up. Overall, CCB are preferred for epicardial spasms, but diltiazem does not effectively treat microvascular spasms; the effects of other CCB on microvascular spasms are unknown.^24^

##### Myocardial bridging

Non-dihydropyridine CCB are used as second-line therapy for myocardial bridging based on anecdotal evidence from individual case reports.

#### Nitrates

##### CMD

Nitrates have been used in the treatment of angina for their hemodynamic effects on the coronary circulation including arterial, arteriolar, and venous dilation and enhancement of collateral flow.^37^ Naturally occurring NO causes coronary arterial vasodilatation through direct effects on vascular smooth muscle cells.^38^ Nitrates do not play a major role in the management of ANOCA due to CMD. In small RCT of patients with microvascular angina, short-acting nitrates did not improve exercise tolerance, and long-acting isosorbide mononitrate provided only a modest reduction in angina and improvements in QoL.^39^^,^^40^ In a trial of 18 individuals with ANOCA, isosorbide dinitrate was associated with reduced exercise tolerance compared with placebo and a decreased time to ST-segment depressions during stress testing.^41^ In addition to limited efficacy, common LAN side effects include flushing, headaches, and hypotension. LAN should be reserved for patients with ANOCA due to CMD who have refractory angina after trialing first- and second-line therapies.

##### Vasospasm

Short-acting nitroglycerin, typically sublingual, is the only therapy to rapidly treat angina from coronary vasospasm in the acute setting.^6^ Longer-acting nitroglycerin preparations are often reserved as second-line therapy for vasospasm after trialing a combination of 2 CCB. In observational studies, long-term nitrate use for vasospasm is associated with increased risks of adverse cardiovascular events.^42^, ^43^, ^44^ Furthermore, side effects, drug tolerance, and rebound angina with discontinuation make LAN less desirable than CCB for long-term use.^45^

#### Nicorandil

##### CMD

Nicorandil is an antianginal coronary vasodilator that may be beneficial in patients with CMD. Intracoronary (IC) use increases Doppler-derived coronary flow velocity and decreases coronary microvascular resistance.^46^ In a crossover study of 13 patients with ANOCA, the time to ST-segment depression and exercise duration were prolonged after 2 weeks of nicorandil versus placebo.^47^ A meta-analysis of 24 small, predominantly nonrandomized studies evaluating nicorandil in ANOCA also reported prolonged time to ST-segment depression and improved angina symptoms.^48^ Nicorandil is not approved for clinical use by the United States (US) Food and Drug Administration but is available in Europe, Asia, and Canada for the treatment of refractory angina.

##### Vasospasm

Nicorandil has not been studied for coronary vasospasm.

##### Myocardial bridging

Nicorandil has not been studied for patients with myocardial bridges.

#### Ranolazine

##### CMD

Ranolazine improves exercise capacity and reduces angina episodes in individuals with chronic stable angina. In the MARINA pilot trial, which randomly assigned 26 patients with ANOCA to ranolazine or placebo, no significant differences in invasive CFR, angina frequency scores, or Duke Activity Status Index were observed.^49^ Ranolazine did not improve myocardial perfusion reserve index (MPRI) by cardiac magnetic resonance imaging in a placebo-controlled RCT (n = 128; 96% women) with ANOCA.^50^ In a randomized, double-blind, placebo-controlled crossover trial enrolling 20 women with ANOCA and refractory symptoms, 4 weeks of ranolazine improved QoL, physical functioning, and angina stability versus placebo.^51^ In the WISE (Women’s Ischemia Syndrome Evaluation) randomized crossover trial enrolling symptomatic patients with ANOCA and decreased MPRI, participants with reduced CFR <2.5, but not those with preserved CFR, had improvements in angina frequency and higher MPRI with ranolazine than placebo.^52^ In the ChaMP-CMD trial, 87 patients with ANOCA underwent blinded CFT and were randomly assigned to ranolazine or amlodipine for 4 weeks. Patients with CMD (CFR <2.5) had greater increments in exercise time compared with those with preserved CFR after treatment with ranolazine and amlodipine. However, only ranolazine improved angina scores in patients with low CFR.

##### Vasospasm

Ranolazine has not been studied in individuals with ANOCA due to vasospasm.

##### Myocardial bridging

Ranolazine has not been studied for patients with myocardial bridges.

### Nonantianginal pharmacotherapy for ANOCA

#### Angiotensin enzyme inhibitors

##### CMD

Angiotensin-converting enzyme inhibitors block the breakdown of bradykinin and, consequently, vasodilatory prostaglandins and NO. ACEi play a potential role in the treatment of ANOCA due to CMD. In hypertensive patients with ANOCA, long-term treatment with enalapril increased CFR.^53^ In a small, placebo-controlled, double-blind crossover study of enalapril 10 mg daily in 10 patients with ANOCA and reduced CFR, ACEi prolonged exercise duration and the time to ST-segment depression.^54^ In a separate placebo-controlled RCT of 20 participants with ANOCA, enalapril improved exercise duration, decreased angina frequency, and increased CFR by IC Doppler.^55^ The combination of ACEi (ramipril 10 mg daily) and atorvastatin reduced angina and improved exercise duration and flow-mediated dilation (FMD) of the brachial artery in a placebo-controlled randomized trial enrolling 45 participants with ANOCA.^56^

Perhaps the most compelling study to demonstrate the benefit of ACEi in patients with CMD was the WISE substudy of 61 women with ANOCA and invasive CFR <3.0; participants were randomized to receive quinapril 80 mg daily or placebo for 16 weeks.^57^ Quinapril reduced angina frequency and improved Doppler-derived CFR in patients with a baseline CFR<2.5 (Δ CFR >0.4 in 62% with ACEi versus 32% with placebo).^57^ Although quinapril is no longer manufactured, it is assumed that the benefits of ACEi in CMD apply to the entire drug class.^58^

Whether ARB have equivalent effects on coronary microcirculatory function as ACEi remains uncertain. In a small study of patients referred for percutaneous coronary intervention (PCI) who were free of CAD in the left anterior descending artery (LAD), Doppler-derived CFR in the LAD was improved in patients randomly assigned to receive 6 months of candesartan versus placebo.^59^ However, studies involving asymptomatic cohorts suggest that ARB may be less effective than ACEi in improving CFR and myocardial blood flow. Unlike ACEi, ARB do not potentiate endogenous bradykinin or increase NO. Neprilysin inhibitors (eg, sacubitril) block bradykinin degradation but have not yet been studied in the treatment of ANOCA due to CMD.

##### Vasospasm

There is limited evidence to support ACEi or ARB in vasospasm.

##### Myocardial bridging

There is no evidence to support ACEi or ARB for this condition.

#### Lipid-lowering agents

##### CMD

Statins reduce LDL cholesterol, have pleiotropic antiinflammatory effects, and can improve CFR, but clinical outcomes data in patients with ANOCA are limited.^60^, ^61^, ^62^, ^63^ Statins improved QoL, time to ST-segment depression, and exercise times in several small placebo-controlled RCT of patients with ANOCA.^56^^,^^64^^,^^65^ However, rosuvastatin did not improve invasive measures of microvascular function in a placebo-controlled RCT of 66 women with ANOCA.^66^ Nonstatin lipid-lowering agents have not been studied for CMD. Proprotein convertase subtilisin/kexin 9 inhibitors have not yet been studied in the treatment of ANOCA.

##### Vasospasm

Statins improve endothelial function and therefore are recommended in patients with vasospasm and endothelial dysfunction.^7^

#### Antiplatelet therapy

The role of antiplatelet therapy in patients with ANOCA is being investigated. In a small study that enrolled 31 individuals with ANOCA, the time to platelet aggregation was shorter in ANOCA patients compared with those with obstructive CAD or healthy controls.^67^ Although intriguing, the study did not evaluate microvascular function, and the mechanism of increased platelet aggregation at rest in these patients remains uncertain.

In a study of patients with acute coronary syndrome undergoing PCI, intensive antiplatelet therapy with ticagrelor appeared to reduce CMD compared with clopidogrel.^68^ In ANOCA, low-dose aspirin is commonly recommended despite observational evidence suggesting associations between aspirin and coronary vasospasm,^69^ and the well-established bleeding risks. In the CorMicA trial, 70% received aspirin at follow-up.^6^ In ANOCA patients with angiographic evidence of epicardial atherosclerosis and concomitant CMD, antiplatelet drugs may be indicated for primary or secondary prevention irrespective of effects on the microcirculation. However, the benefit of aspirin in patients with CMD in the absence of atherosclerosis is unknown.

#### Glycemic control therapy

Several agents approved for glycemic control may benefit patients with CMD. In a double-blind RCT enrolling 33 nondiabetic women with ANOCA, twice daily metformin reduced the incidence of chest pain, improved Duke treadmill scores, and enhanced endothelium-dependent microvascular function in the peripheral arteries as measured by laser Doppler imaging compared with placebo.^70^ In 10 patients with CAD undergoing PCI of the LAD, glucagon-like peptide-1 (GLP-1) increased invasively measured resting coronary flow velocity and decreased basal microcirculatory resistance compared with placebo, although GLP-1 did not affect hyperemic pressure or flow velocity indexes.^71^ GLP-1 receptor agonists can dramatically lower body weight and may independently improve anginal symptoms. Sodium-glucose cotransporter 2 inhibitors, such as empagliflozin, have been associated with improved peripheral vascular endothelial function as measured by brachial artery FMD^72^ but not other vascular measures.^72^^,^^73^

### Other pharmacotherapy for ANOCA

#### L-arginine

L-arginine, a precursor of endothelial-derived NO, improves coronary microvascular responses to Ach.^74^^,^^75^ A 6-month placebo-controlled RCT of L-arginine 3 g 3 times daily in 26 individuals with ANOCA revealed that long-term L-arginine decreased serum endothelin concentrations and improved chest pain to a greater extent than placebo.^74^ In 11 patients at risk for endothelial dysfunction, L-arginine infusion attenuated abnormal increases in coronary vascular resistance to cold pressor testing.^76^ However, in a study of women with ANOCA (n = 12), the infusion of L-arginine did not improve myocardial blood flow at rest or in response to cold pressor testing.^77^ L-arginine oral therapy can be considered in patients with endothelial-dependent CMD as measured by reduced CBF during Ach provocation.

#### Ivabradine

Ivabradine reduces heart rate and prolongs diastole without diminishing myocardial contractility or blood pressure, increases resting CBF, and may inhibit the formation of reactive oxygen species.^78^^,^^79^ Data supporting the use of ivabradine in patients with ANOCA are limited. In a trial of 46 adults with ANOCA, CFR< 2.5 by Doppler echocardiography, and suboptimal angina control, 4 weeks of ivabradine 5 mg twice daily and ranolazine 375 mg twice daily improved angina and decreased anginal frequency compared with placebo, though ranolazine was associated with more favorable effects on QoL compared with ivabradine.^80^ Unfortunately, neither ivabradine nor ranolazine increased CFR in response to adenosine.

#### Phosphodiesterase inhibitors

##### CMD

An ancillary study from WISE evaluated the role of phosphodiesterase (PDE) type-5 inhibition in 23 symptomatic women with ANOCA, with CFR measured before and 45 minutes after 100 mg oral sildenafil. Sildenafil improved CFR, with the greatest impact among patients with the most abnormal reduced flow reserves.^81^ However, long-term PDE-5 inhibition has not been tested in patients with ANOCA due to CMD, and its net clinical benefit is unknown.

##### Vasospasm

Cilostazol, a PDE type-3 inhibitor, reduces angina frequency and severity in patients with vasospasm refractory to CCB and nitrates.^82^^,^^83^ Cilostazol is also associated with increased CFR and coronary volumetric flow in patients with Ach-provoked vasospasm.^84^ In an RCT enrolling 40 individuals with vasospasm, long-acting cilostazol 200 mg once daily and isosorbide mononitrate 20 mg twice daily provided similar reductions in angina, but cilostazol was associated with a lower incidence of headaches or dizziness.^85^ Cilostazol can be considered a third-line agent in cases of refractory vasospasm.

#### Fasudil

##### Vasospasm

Fasudil is a Rho-associated protein kinase inhibitor with favorable effects in the setting of vasospasm that may potentiate coronary vasodilation in response to nitrates.^86^, ^87^, ^88^ In an RCT enrolling 20 patients with epicardial vasospasm, IC fasudil, but not saline, attenuated spasm during Ach provocation.^89^ In 18 patients with microvascular spasms, IC fasudil pretreatment also reduced ischemic responses to Ach provocation.^90^ Finally, among patients with ANOCA, 30 mg IC fasudil decreased IMR in the subgroup of patients with a baseline IMR >18 and vasospasm in response to Ach.^91^ Data evaluating longer-term clinical outcomes of fasudil in ANOCA are limited. Fasudil is not available in the US.

#### Trimetazidine

There is limited and contradictory evidence regarding the benefit of trimetazidine for CMD. In a crossover, RCT enrolling 16 patients with ANOCA, trimetazidine did not improve anginal episodes or the time to ST-segment depression compared with placebo.^92^ However, in 2 other placebo-controlled RCT enrolling patients with ANOCA, angina was improved and total exercise time and time to ST depressions were prolonged after trimetazidine treatment.^93^^,^^94^ Trimetazidine is not available in the US.

#### Adenosine antagonists

The phenomenon of adenosine hypersensitivity or “myocardial migraine” reflects abnormal nociception to adenosine and, in some patients, may be responsible for angina in ANOCA.^95^ In individuals with ANOCA, oral aminophylline, a nonselective adenosine receptor antagonist, decreased anginal frequency and prolonged time to angina.^96^^,^^97^ Other oral adenosine receptor antagonists, such as caffeine, have not been tested in patients with ANOCA.

#### Antidepressants

Tricyclic antidepressants (TCA) and selective serotonin reuptake inhibitors (SSRI) improve angina in patients with ANOCA through modulation of cardiac nociception.^98^ In a meta-analysis of trials, antidepressant therapy improved angina in patients with ANOCA.^99^ Low-dose TCA (imipramine 50 mg daily) improved QoL and reduced chest pain episodes by 50% in patients with ANOCA.^100^^,^^101^ SSRI have fewer adverse drug reactions and are better tolerated than TCA. In a placebo-controlled RCT of SSRI therapy in ANOCA, sertraline reduced the severity of angina.^102^ SSRI and TCA are reasonable for use in the management of patients with ANOCA who have symptoms despite conventional antianginal therapy.

#### Hormone replacement therapy

Hormone replacement therapy remains controversial in the management of menopausal women with cardiovascular disease. In a study of 35 postmenopausal women with ANOCA who were randomly assigned to low-dose norethindrone/ethinyl estradiol for 12 weeks versus placebo, estrogen improved angina, but no differences in brachial FMD or myocardial ischemia were observed.^103^ In a double-blind RCT enrolling 56 postmenopausal women with angina, 6 weeks of estrogen/progestin combination (17β-estradiol–drospironene) increased myocardial perfusion reserve compared with placebo.^104^ Among men with stable angina, 12 weeks of 5 mg daily transdermal testosterone prolonged exercise time to 1-mm ST-segment depression versus placebo.^105^

#### Anti-inflammatory therapy

Anti-inflammatory medications hold promise to treat CMD and improve endothelial function, but there are no studies specifically evaluating ANOCA patients. In a meta-analysis of 20 trials enrolling individuals with rheumatoid arthritis, psoriasis, or psoriatic arthritis, tumor necrosis factor-alpha blockers were associated with improved peripheral vascular endothelial function.^106^ Among patients with rheumatoid arthritis, the interleukin-1 receptor antagonist anakinra, and an interleukin-6 receptor blocker tocilizumab, improved echocardiographic Doppler-derived measures of CFR.^107^ More recently, among patients with ST-elevation myocardial infarction, tocilizumab resulted in less extensive microvascular obstruction compared with placebo.^108^

#### Cyproheptadine

##### Vasospasm

In small case studies, cyproheptadine, a nonselective serotonergic antagonist traditionally used as an antiallergic agent, has been reported to treat vasospasm.^56^ Cyproheptadine blocks the vasoconstrictive effects of serotonin and can be considered when a refractory spasm has failed to respond to a combination of CCB and LAN.

### Medications without supporting evidence in ANOCA

#### Endothelin antagonists

Endothelin-1 is a circulating vasoconstrictor that may play a role in the pathophysiology of CMD.^109^ Zibotentan, an oral endothelin A receptor antagonist, was studied in the PRIZE trial that enrolled 118 patients with ANOCA due to probable or definite microvascular angina.^109^^,^^110^ The study was enriched to ensure that 50% of participants had a single nucleotide polymorphism associated with higher endothelin-1 expression. Participants were randomized to either zibotentan 10 mg daily or placebo for 12 weeks, with treadmill exercise duration as the primary outcome. There was no significant difference in exercise duration with zibotentan versus placebo (–4.26 seconds; 95% CI, –19.60 to 11.06; *P* = .59), and adverse events were more common with zibotentan (60.2% vs 14.4%, *P* < .001).^111^ Given these disappointing results, the future of endothelin antagonism in the treatment of CMD is uncertain.

#### Alpha-blockers, aldosterone inhibitors, and allopurinol

Current evidence does not support the use of these agents to reduce angina and improve endothelial function or CFR in patients with ANOCA.

## Device-based and emerging novel therapies

### Enhanced external counter-pulsation

Noninvasive enhanced external counter-pulsation (EECP) enhanced endothelial function in a randomized, sham-controlled study of patients with stable ischemic heart disease^112^ and improved microvascular function after coronary stent implantation.^113^ In an observational study of patients with ANOCA, EECP was associated with significant improvements in angina burden, 6-minute walk test, and functional status.^114^ EECP also resulted in increased CFR in patients with ANOCA^115^ and improved coronary flow in patients with coronary slow flow syndrome.^116^ EECP is currently approved for the treatment of patients with CCS class 3 to 4 angina despite maximum medical therapy.

### Coronary sinus reducer

The coronary sinus reducer (CSR) increases coronary sinus pressures and enhances myocardial perfusion through flow redistribution and has been associated with angina improvement in patients with refractory angina from obstructive CAD.^117^^,^^118^ In a phase II trial enrolling 30 patients with ANOCA due to CMD, CSR implantation increased CFR and improved CCS angina class and Seattle Angina Questionnaire scores at 120 days postimplantation.^119^ In a small observational study of 24 patients with obstructive CAD who underwent CFT before and 4 months after CSR implantation, IMR decreased, CFR increased, and angina class and disease-related QoL improved after CSR.^120^ Also, in a study of 20 patients with CMD, balloon occlusion of the coronary sinus led to decreases in resting and hyperemic coronary resistance.^121^ The ongoing COSIMA and COSIRA II trials will provide insights into the effectiveness of CSR therapy in patients with angina due to CMD.

### CD34+ cell delivery and gene therapy

Autologous bone marrow–derived endothelial progenitor cells (CD34+ cells) can enhance angiogenesis and vascular repair. Intramyocardial delivery of autologous CD34+ cells improves angina frequency and exercise time compared with placebo in patients with refractory angina from obstructive CAD.^122^ In a 2-center, phase I study (ESCaPE-CMD) evaluating the feasibility and safety of autologous CD34+ stem cells in patients with ANOCA and CMD, cell therapy improved CFR, angina frequency, angina class, and QoL.^123^ A phase II, double-blind, placebo-controlled RCT to evaluate CD34+ cell therapy for CMD was stopped prematurely by the sponsor due to slow enrollment. Gene therapy is another potential avenue of therapy for CMD. Adenoviral vectors expressing proangiogenic isoforms of the vascular endothelial growth factor are currently in development for refractory angina and require further study in ANOCA.

### Surgical coronary unroofing

For patients with myocardial bridges who fail medical management and have an intolerable QoL, surgical myotomy (unroofing) can significantly improve SAQ scores.^124^ Stenting and coronary artery bypass grafting are discouraged because of safety issues and lack of efficacy data, respectively.

### Nerve stimulation

Transcutaneous nerve stimulation has beneficial effects on angina through central nervous system pain pathway modulation.^125^ Transcutaneous nerve stimulation reduced the frequency of symptoms and improved myocardial perfusion on nuclear positron emission tomography in patients with ANOCA.^126^ There is not enough evidence to recommend its use in patients with refractory angina and ANOCA due to CMD.

## Practical implications for clinical care: Initial therapy and follow-up

There are no definitive guidelines for the treatment of ANOCA, but management should be tailored to the results of CFT (Tables 1 and 2). Sequential initiation and careful up-titration of medical therapy every few weeks is typically preferred to evaluate the effectiveness and ensure patient compliance and tolerance, including side effects and potential overlap between endotypes may require careful selection of individual agents (Table 3, Central Illustration). Multidisciplinary care teams with a general cardiologist who can titrate medical therapies for angina, blood pressure, and lipid control, an invasive cardiovascular disease specialist with expertise in CFT, and primary care, and a nonphysician advanced practice providers who can address glycemic control, antidepressant use, and weight loss considerations are essential. Psychologists are integral in addressing the anxiety and stress related to ANOCA.

**Table 1:** Therapies for ANOCA by diagnosis.

Therapy	Coronary microvascular dysfunction; Ado CFR < 2-2.5 and/or IMR ≥ 25 / HMR ≥ 2-2.5	Coronary spasm >90% narrowing Ach; endothelial dysfunction >0%-90% Ach; microvascular spasm Ach pain + ECGΔ	Myocardial bridging; Dobu RFR ≤0.76
Lifestyle interventions	Exercise and weight loss	Exercise and weight loss	
Medical therapy	Antianginal therapy, beta-blockers, calcium channel blockers, ranolazine, nicorandil^a^	Calcium channel blockers, short-acting nitrates, long-acting nitrates	Beta-blockers, calcium channel blockers
Nonantianginal pharmacotherapies	Angiotensin-converting enzyme inhibitors, statins, antiplatelet therapies^b^, metformin/glucagon-like peptide-1	Angiotensin-converting enzyme inhibitors, statins, metformin	Ivabradine
Other therapies	L-arginine, ivabradine, PDE-5 inhibition, trimetazidine,^a^ adenosine antagonists, TCA/SSRI antidepressants, hormone therapy	Cilostazol, fasudil^a^, cyproheptadine	
Device-based/surgical therapy	Enhanced external counter-pulsation, coronary sinus reducer therapy, transcutaneous nerve stimulation		Coronary artery unroofing
Cell therapy	CD34+ cell-based therapies		

Ach, acetylcholine; Ado, adenosine; ANOCA, angina with nonobstructive coronary arteries; CFR, coronary flow reserve; Dobu, dobutamine; ECGΔ, electrocardiogram changes; HMR, hyperemic microvascular resistance; IMR, index of microcirculatory resistance; PDE, phosphodiesterase; RFR, resting full-cycle ratio; SSRI, selective serotonin reuptake inhibitors; TCA, tricyclic antidepressant.

a Not FDA approved for clinical use in the United States.

b Despite no evidence of a net clinical benefit, aspirin is routinely prescribed to patients with ANOCA.

**Table 2:** Treatment approaches based on abnormality identified by functional pathway.

Treatment class	Coronary microvascular dysfunction	Coronary vasospasm, endothelial dysfunction, microvascular spasm	Myocardial bridging
ACEi/ARB	↑ CFR, improves microvasculature remodeling, improves endothelial vasomotor function		
Statins	↑ CFR, improves endothelial function; pleiotropic effects (reduced vascular inflammation)	↑ CFR, improves endothelial function; pleiotropic effects (reduced vascular inflammation)	
Beta blocker	↓ myocardial oxygen consumption	(Avoid beta-1 selective BB)	↓ myocardial oxygen consumption, ↓ contractility
Calcium channel blocker	Vascular smooth muscle relaxation; ↓ myocardial oxygen consumption	↓spontaneous and inducible coronary vasospasm via vascular smooth muscle relaxation ↓ oxygen demand	(Non-DHP CCB) ↓ myocardial oxygen consumption, ↓ contractility
Nitrates	Limited benefit/avoid use	↓spontaneous and inducible coronary vasospasm via epicardial vasodilation, ↓ oxygen demand	Limited benefit
Ranolazine	Improves MPRI in CMD		
Nicorandil^a^	K channel agonist-coronary microvasculature effect		
Exercise training	↑ CFR		
Device-based/surgical therapy	Coronary sinus reducer redistributes blood flow		Surgical unroofing improves myocardial blood supply during systolic and diastolic coronary flow.

ACEi, angiotensin-converting enzyme inhibitor; ARB, angiotensin receptor blockers; CCB, calcium channel blocker; CFR, coronary flow reserve; DHP, dihydropyridine; MPRI, myocardial perfusion reserve index.

a Not FDA approved for clinical use in the United States.

**Table 3:** Treatment recommendations for ANOCA with side effects.

Treatment class	Disease	Treatment options	Side effects
CCB (non-DHP ± DHP)	Spasm	Verapamil 80 mg twice a day, verapamil SR 120-360 mg daily, Diltiazem 30 - 60 mg three times a day, diltiazem SR 120-360 mg, amlodipine 2.5-10 mg daily, nifedipine 30-90 mg daily	Hypotension, bradycardia, combination of non-DHP ± DHP can be used in bradycardia or refractory spasm
Beta blockers	CMD, myocardial bridging	Metoprolol 25-400 mg daily, bisoprolol 2.5-10 mg daily, nebivolol 2.5-40 mg daily, carvedilol 3.125-25 mg twice a day	Bradycardia, hypotension, may worsen coronary spasm, raynaud, fatigue, nightmares, erectile dysfunction
Vasodilatory beta blockers	Spasm, endothelial dysfunction, CMD	Nebivolol 2.5-40 mg daily, carvedilol 3.125-25 mg, twice a day	Hypotension, nausea, diarrhea
Nitrates	Spasm, microvascular spasm	ISMN 30-120 mg daily, NTG patch 0.2 mg/h to 0.8 mg/h, SL NTG 0.4 mg as needed	Tolerance and tachyphylaxis, headaches and migraines, hypotension, worsening systolic compression of the myocardial bridge
ACEi/ARB	CMD, endothelial dysfunction	Lisinopril 2.5-40 mg daily or dose given twice a day, losartan 25-100 mg daily	Hypotension Cough with ACEi Hyperkalemia Worsening renal function
Ranolazine	CMD, microvascular spasm	Ranolazine 500-1000 mg twice a day	QTc prolongation interaction with CCB, SSRI, and ivabradine
Pacemaker current (If) inhibitor	CMD	Ivabradine 5-7.5 mg twice a day	Bradycardia in combination Prolonged QTc
L-arginine	CMD, spasm	L-arginine (up to 4.5 g twice a day) powder or pill	Contraindicated in prior MI, nausea and indigestion
PDE-5 inhibitors	CMD	Sildenafil 25 mg twice a day, tadalafil 10 mg twice a day	Hypotension with NTG
PDE-3 inhibitors	Spasm	Cilostazol 50-100 mg twice a day	Headache, diarrhea, edema
Tricyclic antidepressants	Abnormal nociception	Imipramine 50 mg daily	QTc and QRS prolongation, dry mouth
Enhanced external counter-pulsation	CMD, endothelial dysfunction	7 weeks cycles with 5 days a week 1 to 2 hour sessions	Limited access, dedicated centers for the treatments

ACE, angiotensin-converting enzyme inhibitor; ARB, angiotensin receptor blockers; CCB, calcium channel blocker; CMD, coronary microvascular dysfunction; DHP, dihydropyridine; MI, myocardial infarction; NTG, nitroglycerin; PDE, phosphodiesterase; SSRI, selective serotonin reuptake inhibitors.

**Central Illustration:**
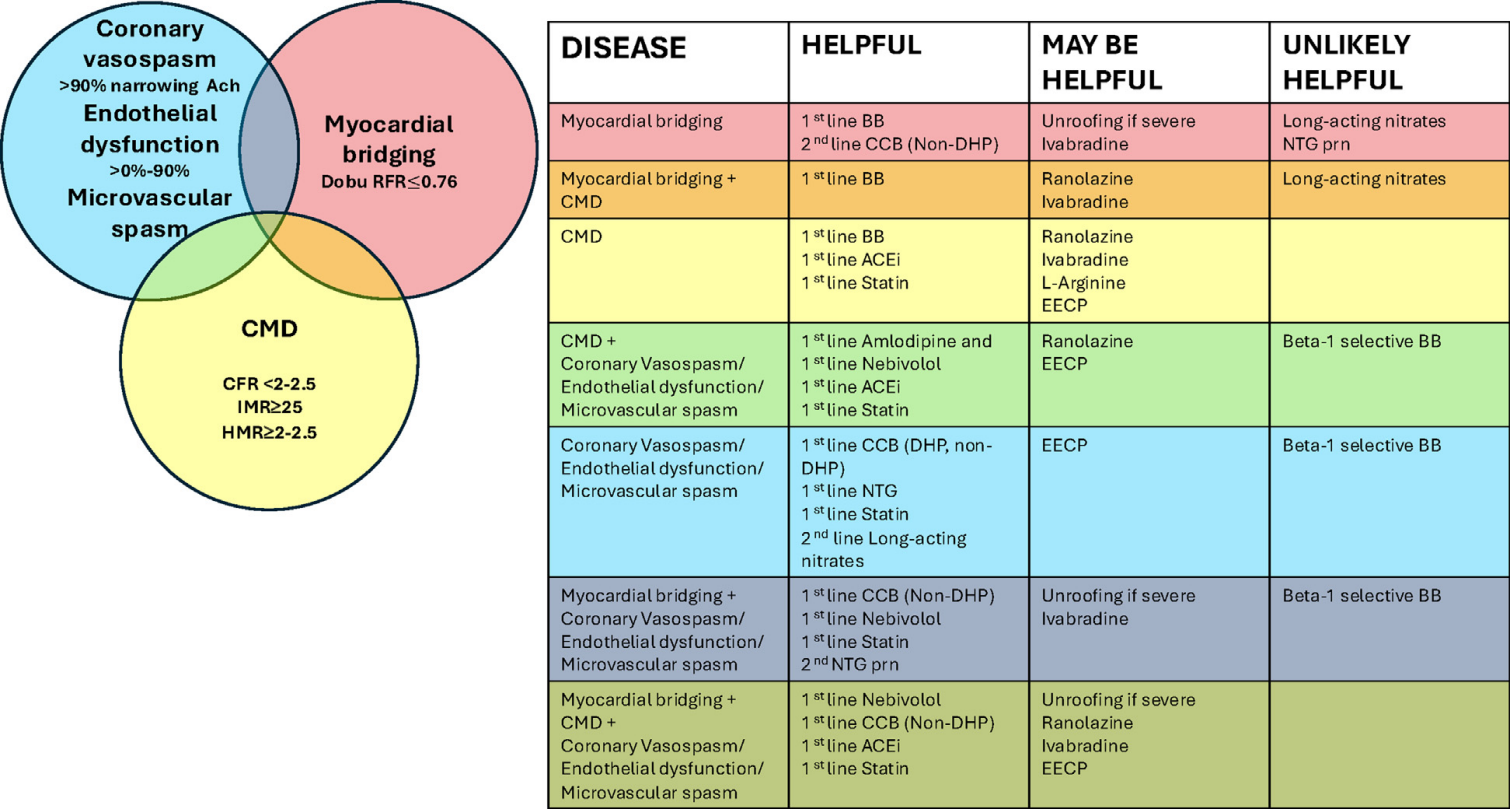
**Main angina with nonobstructive coronary arteries diagnoses with overlap and potential combination therapies.** ACEi, angiotensin-converting enzyme inhibitor; Ach, acetylcholine; BB, beta blocker; CCB, calcium channel blocker; CFR, coronary flow reserve; CMD, coronary microvascular dysfunction; DHP, dihydropyridine; Dobu, dobutamine; EECP, enhanced external counter-pulsation; HMR, hyperemic microvascular resistance; IMR, index of microcirculatory resistance; NTG, nitroglycerin; RFR, resting full-cycle ratio.

## Areas of uncertainty

Treatment studies for ANOCA are small and include heterogeneous patient cohorts. Few include invasive CFT to diagnose specific ANOCA endotypes or have serial CFT.^7^^,^^8^ Heterogeneity in assessment techniques (eg, bolus thermodilution vs invasive Doppler) and variable cutoffs complicate comparisons between studies. Patient-reported outcome measures, such as QoL and angina severity questionnaires, are subjective and may be affected by unmeasured confounders. Future studies should attempt enrolling large cohorts of individuals with ANOCA based on invasive CFT and randomly assign them to study drug(s) versus placebo, with long-term follow-up for angina and clinical outcomes including the following: cardiovascular death, MI, stroke, and heart failure along with effects on QoL, emergency and hospital readmissions for angina (Supplemental Table S1).

## Conclusion

Coronary microvascular dysfunction, vasospasm, and myocardial bridging are important causes of ANOCA. The treatment of ANOCA should be tailored according to the underlying endotype identified through CFT. However, few high-quality treatment trials exist. Large multicenter RCT of therapies are needed to guide the management of patients with ANOCA.
