# Supplementary material for: Current Evidence-Based Treatment of Angina With Nonobstructive Coronary Arteries (ANOCA)
Source: J Soc Cardiovasc Angiogr Interv. 2025 Apr 15;4(7):102633. doi: 10.1016/j.jscai.2025.102633 (PMC12418408; doi:10.1016/j.jscai.2025.102633)
Supplement: Supplemental Table S1 [file mmc1.docx]

Table 4. Ongoing clinical trials for ANOCA therapies

| Trial name | Population  Methods | Intervention | Comparison | Outcomes |
| --- | --- | --- | --- | --- |
| COSIMA: COronary SInus Reducer for the Treatment of Refractory Microvascular Angina  (COSIMA)  Randomized, controlled,  parallel-groups trial  ClinicalTrials.gov ID NCT04606459 | ANOCA:  CFT to identify CMD | Guideline-directed optimal medical therapy with coronary sinus Reducer (Neovasc Reducer™ System) implantation | Guideline-directed medical therapy alone | QoL questionnaires   - SAQ   Change in CCS class angina  Safety outcomes for CSR  Adverse events:  Death (all cause and cardiovascular) Myocardial infarction Revascularization Periprocedural complications |
| The Impact of Vericiguat on Microvascular Function in Patients With Documented Vasospastic Angina Pectoris (ViVA)  Randomized, crossover placebo controlled trial  ClinicalTrials.gov ID NCT06415227 | ANOCA:  **Vasospasm:** coronary epicardial and microvascular  **Vericiguat**: soluble guanylate cyclase stimulator directly acting on the NO signaling from the endothelium to vascular smooth muscle cells | **Vericiguat** 2.5 mg, 5 mg and 10 mg) first; placebo second | Placebo first;  **Vericiguat** 2.5 mg, 5 mg and 10 mg second | Microvascular function assessed with LASCA : Peak cutaneous microvascular conductance during acetylcholine iontophoresis at 10 and 20 weeks  QoL questionnaires   - SAQ |
| Coronary Sinus Reducer Implantation in Patients With Ischaemia and Non-obstructed Coronary Arteries and Coronary Microvascular Dysfunction. (REMEDY-PILOT)  Randomized, double-blinded, sham-controlled pilot study with parallel arms of participants randomized to CS Reducer or sham procedure  ClinicalTrials.gov ID NCT05492110 | ANOCA:  CFT at baseline  CMR with Ado | Neovasc coronary sinus reducer implantation | Sham-procedure | QoL questionnaires   - SAQ   Change in CCS class angina  Safety outcomes for CSR  6 months CMR change in absolute myocardial perfusion |
| Efficacy of Targeted Medical Therapy in Angina and Nonobstructive Coronary Arteries (MVP-ANOCA)  Randomized Parallel Assignment, placebo controlled trial  ClinicalTrials.gov ID NCT06424834 | ANOCA:  **Vasospasm:** coronary epicardial and microvascular  **CMD**  **Myocardial bridge**  **Mixed:** spasm/CMD/ MB | **Vasospasm:** Amlodipine 2.5- 10 mg  **CMD:** Nebivolol 5-20 mg  **Myocardial bridge:** Nebivolol: 5-20 mg  **Mixed:**  Amlodipine 2.5mg initial dose, 10mg max dose; plus Nebivolol 5mg initial dose, 20mg max dose | Placebo | QoL questionnaires   - SAQ   MACE: cardiac death, myocardial infarction, and hospital presentation for unstable angina  Safety endpoints CFT procedure: bleeding, MI, dissection, arrhythmia non self limiting |
| Exercise and Coronary Microvascular Disease (ExerciseCMD)  ClinicalTrials.gov ID  NCT05810051 | ANOCA:  CMD | OMT | OMT plus a program of cardiac rehabilitation | QoL questionnaires   - SAQ   VO2 max  Vessel volume by coronary CTA |
| Stratified Medicine in Angina  (iCorMicA)  Multicenter, prospective, randomized, double-blind, sham-controlled, parallel-group, end-point trial and registry  ClinicalTrials.gov ID NCT04674449 | ANOCA:  CMD stratified medical therapy guided by CFT:  Targeted therapy according to CFT diagnosis blinded  randomization (1:1) | CFT:  Measurement of CFR, IMR and vasoreactivity with Ach  CFT-guided, results disclosed | Sham procedure  blinded control CFT undertaken but results not disclosed, standard of care group | QoL questionnaires   - SAQ   Patient Reported Outcome Measures (PROMS)  EQ-5D-5L,  Illness perception (Brief IPQ), Treatment satisfaction(TSQM), Duke Activity Status Index(DASI), the International Physical Activity Questionnaire (IPAQ-SF)  MACE and by groups  Safety of CFT |
| Nebivolol ANOCA Treatment Randomized Trial (MOTORS)  Randomized parallel placebo controlled  ClinicalTrials.gov ID NCT06755801 | ANOCA with CMD:  CFR<2.0 and/or IMR ≥ 25 | Nebivolol 5mg daily, with dose increases up to 20 mg daily as tolerated | Placebo | QoL questionnaires   - SAQ   MACE: cardiac death, myocardial infarction, and hospital presentation for unstable angina, stroke or heart failure requiring hospitalization  ETT Bruce protocol |
| Study Targeting Myocardial Perfusion and Symptom Relief in Women with SGLT2 Inhibitors (STRONG)  Single-center, randomized, double-blind, placebo-controlled study  ClinicalTrials.gov ID NCT06600178 | ANOCA:  Women  CMD  coronary flow reserve by CMR at baseline and at 12 weeks | SGLT2i  Dapagliflozin 10 mg | Placebo | QoL questionnaires  -SAQ  -EQ-5D-3L  -Duke Activity  Change in coronary flow reserve by CMR at 12 weeks |
| Transcutaneous Electrical Nerve Stimulation in Patients With Angina and Non-Obstructive Coronary Arteries (TENS-ANOCA)  Single arm study  ClinicalTrials.gov ID NCT06401291 | ANOCA:  Persistent angina despite OMT:  **CMD:** BB, CCB, nicorandil and/or ivabradine  **Vasospasm:** CCB, LAN, and/or nicorandil. In the maximum tolerated dose. | Transcutaneous Electrical Nerve Stimulation for 1 month |  | QoL questionnaires   - SAQ   Change in CCS class angina  Safety endpoints: side effects from TENS  At baseline and at 1 month |

Ach: Acetylcholine; Ado: Adenosine; ANOCA: Angina with Open Coronary Arteries; BB: Beta Blocker; CCS: Canadian Cardiovascular Society; CFR: Coronary Flow Reserve; CSR: Coronary Sinus Reducer; Dobu: Dobutamine; ECG: Electrocardiogram; ETT: Exercise Treadmill Test; HMR: Hyperemic Microvascular Resistance; IMR: Index of Microcirculatory Resistance; ACE: Angiotensin Converting Enzyme inhibitor; ARB: Angiotensin Receptor Blockers; BID: Twice a day; CMR: Cardiac Magnetic Resonance; CCB: Calcium Channel Blocker; CFT: Coronary Function Testing; CMD: Coronary Microvascular Dysfunction; CTA: Computed Tomography Angiogram; DHP: Dihydropyridine; LAN: Long Acting Nitrates; MI: Myocardial Infarction; Non-DHP: Non- Dihydropyridine; NTG: Nitroglycerin; OMT: Optimal Medical Therapy; PDE: Phosphodiesterase Inhibitor; SAQ: Seattle Angina Questionnaire; SGLT2i: Sodium-Glucose Cotransporter 2 inhibitors; SSRI: Selective Serotonin Reuptake Inhibitors; TENS: Transcutaneous Electrical Nerve Stimulation
